# Supplementary material for: Collateral Sensitivity to Fosfomycin of Tobramycin-Resistant Mutants of Pseudomonas aeruginosa Is Contingent on Bacterial Genomic Background
Source: Int J Mol Sci. 2023 Apr 7;24(8):6892. doi: 10.3390/ijms24086892 (PMC10138353; doi:10.3390/ijms24086892)
Supplement: Supplementary file 1 [file ijms-24-06892-s001.zip › ijms-2242043-supplementary.pdf]

Table S1. Concentration of tobramycin (close to MIC) that hindered -but allowed- the growth of each *P. aeruginosa* clinical isolate in glass tube (μg/ml).

ISOLATE ID	ORIGIN	ST	TOBRAMYCIN concentration (μg/ml) used in the ALE experiments
**AND04-004A**	Tracheal aspirate	244	0.4
**ARA02-005**	Blood	267	0.3
**BAL02-001**	Sputum	1619	0.2
**BAL04-002**	Blood	1816	0.3
**CAN01-002**	Sputum	111	0.4
**CAN01-003**	Sputum	111	6
**CAT02-004**	Blood	244	0.2
**CAT06-005**	Sputum	175	6
**CAT09-004**	Blood	244	0.3
**CLE03-004**	Tracheal aspirate	381	0.5
**CLM01-003**	Sputum	709	0.3
**CVA01-006**	Sputum	175	12
**EXT01-004**	Sputum	3353	0.8
**GAL01-001**	Sputum	560	0.2
**GAL02-004**	Sputum	217	2
**ICA01-004**	Sputum	698	0.2

**MAD04-002**	Sputum	242	0.3
**MAD05-009**	Sputum (CF)	27	0.2
**FQSE03-1212-2**	Sputum (CF)	274	0.4
**FQSE10-0503**	Sputum (CF)	274	0.1
**FQSE110603**	Sputum (CF)	701	0.3
**FQSE15-0803**	Sputum (CF)	274	0.4
**FQSE24-0304**	Sputum (CF)	1089	2

ST: sequence type, MIC: Minimal Inhibitory Concentration, CF: cystic fibrosis.

Table S2. MICs in µg/ml of tobramycin and fosfomycin in *P. aeruginosa* clinical strains after evolution in presence or absence of tobramycin.

**AND04-004A**	**parental**	**C1**	**C2**	**C3**	**C4**	**E1**	**E2**	**E3**	**E4**
**TOB**	1.5	1	1.5	1.5	1.5	8	16	8	8
**FOF**	256	256	256	256	256	192	256	48	64
**ARA02-005**	**parental**	**C1**	**C2**	**C3**	**C4**	**E1**	**E2**	**E3**	**E4**
**TOB**	2	2	1.5	2	2	3	6	4	4
**FOF**	96	64	64	64	64	48	96	48	128
**CAN01-002**	**parental**	**C1**	**C2**	**C3**	**C4**	**E1**	**E2**	**E3**	**E4**
**TOB**	1.5	1.5	1.5	1.5	1.5	4	3	4	6
**FOF**	3	3	3	3	3	1.5	3	2	1.5
**CAT02-004**	**parental**	**C1**	**C2**	**C3**	**C4**	**E1**	**E2**	**E3**	**E4**
**TOB**	0.75	0.75	0.75	1	1	6	8	4	4
**FOF**	256	192	256	256	256	32	≥1024	128	128
**CLE03-004**	**parental**	**C1**	**C2**	**C3**	**C4**	**E1**	**E2**	**E3**	**E4**
**TOB**	2	2	3	0.75	2	2	2	6	8
**FOF**	64	96	96	64	32	96	32	32	32
**CVA01-006**	**parental**	**C1**	**C2**	**C3**	**C4**	**E1**	**E2**	**E3**	**E4**
**TOB**	12	16	24	12	32	32	128	128	128
**FOF**	3	12	12	16	12	6	1.5	1.5	2
**MAD05-009**	**parental**	**C1**	**C2**	**C3**	**C4**	**E1**	**E2**	**E3**	**E4**
**TOB**	2	2	2	2	1.5	6	4	4	6
**FOF**	96	96	96	96	128	64	48	48	48
**FQSE10-0503**	**parental**	**C1**	**C2**	**C3**	**C4**	**E1**	**E2**	**E3**	**E4**
**TOB**	1	1	2	1	1.5	4	4	4	4
**FOF**	256	256	256	256	256	96	96	64	96
**FQSE15-0803**	**parental**	**C1**	**C2**	**C3**	**C4**	**E1**	**E2**	**E3**	**E4**
**TOB**	3	2	2	1	3	6	6	4	6
**FOF**	96	96	96	96	96	64	32	32	64
**FQSE24-0304**	**parental**	**C1**	**C2**	**C3**	**C4**	**E1**	**E2**	**E3**	**E4**
**TOB**	1.5	1	0.38	1	1	8	8	12	8
**FOF**	16	24	24	24	24	8	8	12	16
**BAL02-001**	**parental**	**C1**	**C2**	**C3**	**C4**	**E1**	**E2**	**E3**	**E4**
**TOB**	0.75	0.75	0.75	0.75	0.75	4	3	4	6
**FOF**	64	64	48	48	48	48	64	32	48
**BAL04-002**	**parental**	**C1**	**C2**	**C3**	**C4**	**E1**	**E2**	**E3**	**E4**
**TOB**	1	1	1	0.75	1	4	4	6	4
**FOF**	≥1024	≥1024	≥1024	≥1024	768	≥1024	768	768	≥1024
**CAN01-003**	**parental**	**C1**	**C2**	**C3**	**C4**	**E1**	**E2**	**E3**	**E4**
**TOB**	32	32	32	24	32	128	48	96	48
**FOF**	≥1024	≥1024	≥1024	≥1024	≥1024	≥1024	≥1024	≥1024	≥1024

**CAT06-005**	**parental**	**C1**	**C2**	**C3**	**C4**	**E1**	**E2**	**E3**	**E4**
**TOB**	32	129	32	24	32	96	96	96	96
**FOF**	≥1024	≥1024	≥1024	≥1024	≥1024	≥1024	≥1024	≥1024	≥1024
**CAT09-004**	**parental**	**C1**	**C2**	**C3**	**C4**	**E1**	**E2**	**E3**	**E4**
**TOB**	3	2	1.5	3	1	8	4	8	8
**FOF**	64	96	96	128	96	128	96	128	64
**CLM01-003**	**parental**	**C1**	**C2**	**C3**	**C4**	**E1**	**E2**	**E3**	**E4**
**TOB**	2	2	1.5	2	3	3	4	6	24
**FOF**	192	192	192	256	128	128	192	256	256
**EXT01-004**	**parental**	**C1**	**C2**	**C3**	**C4**	**E1**	**E2**	**E3**	**E4**
**TOB**	3	3	3	3	3	12	4	12	4
**FOF**	2	2	2	2	2	2	1 .5	2	1.5
**GAL01-001**	**parental**	**C1**	**C2**	**C3**	**C4**	**E1**	**E2**	**E3**	**E4**
**TOB**	1.5	1.5	2	2	0.5	8	6	6	6
**FOF**	32	24	48	48	256	16	48	24	48
**GAL02-004**	**parental**	**C1**	**C2**	**C3**	**C4**	**E1**	**E2**	**E3**	**E4**
**TOB**	6	6	4	6	4	8	8	8	8
**FOF**	≥1024	≥1024	≥1024	≥1024	≥1024	≥1024	≥1024	≥1024	≥1024
**ICA01-004**	**parental**	**C1**	**C2**	**C3**	**C4**	**E1**	**E2**	**E3**	**E4**
**TOB**	1.5	2	1.5	1.5	1.5	3	4	4	3
**FOF**	48	48	48	32	48	128	128	128	128
**MAD04-002**	**parental**	**C1**	**C2**	**C3**	**C4**	**E1**	**E2**	**E3**	**E4**
**TOB**	3	3	3	3	3	8	8	8	12
**FOF**	6	12	8	8	12	16	12	16	16
**FQSE03-1212-2**	**parental**	**C1**	**C2**	**C3**	**C4**	**E1**	**E2**	**E3**	**E4**
**TOB**	2	4	1.5	1.5	2	4	4	6	4
**FOF**	64	96	64	96	64	96	96	96	96
**FQSE110603**	**parental**	**C1**	**C2**	**C3**	**C4**	**E1**	**E2**	**E3**	**E4**
**TOB**	2	4	2	24	8	6	4	8	6
**FOF**	64	128	256	48	96	96	192	96	128

C1-4: the four replicates evolved in absence of tobramycin. E1-E4: the four replicates evolved in presence of tobramycin. TOB: tobramycin, FOF: fosfomycin.

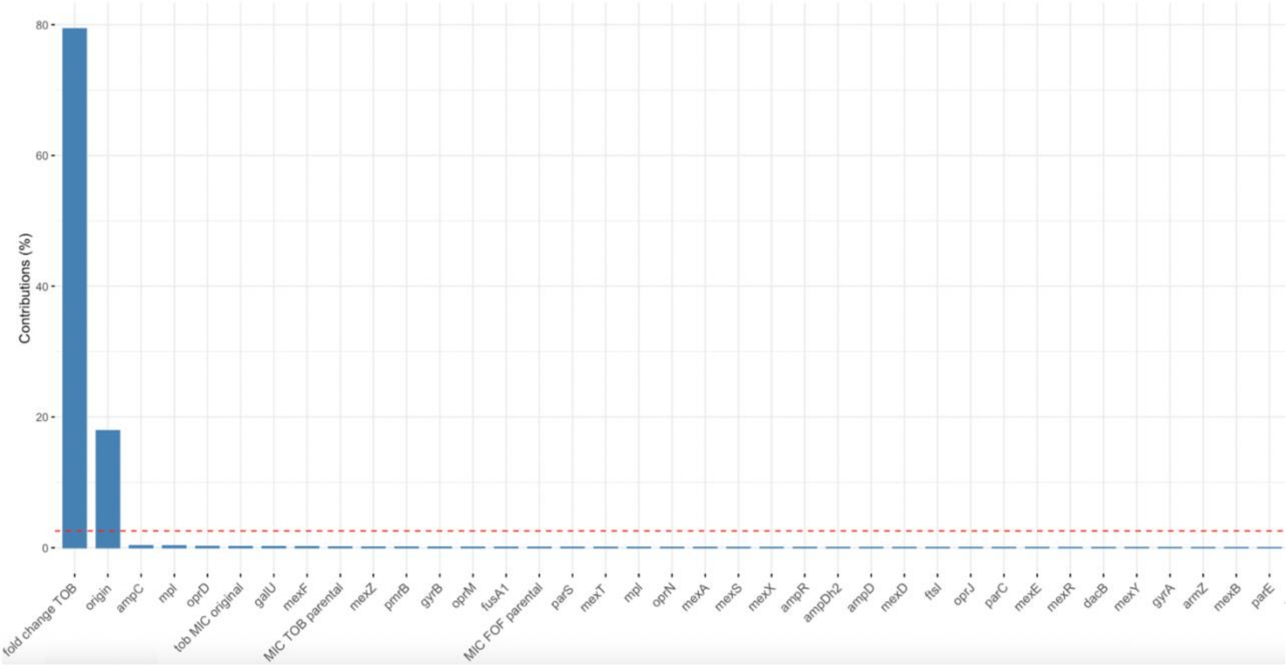

Figure S1. Contribution of different variables to the collateral sensitivity to fosfomycin in *P. aeruginosa* clinical isolates. The variables used for the analysis were: the origin of the strains, their resistome-related mutations (Table 1), their capacity of developing collateral sensitivity to fosfomycin, the MIC of the parental strain to tobramycin and fosfomycin, the fold change of these MICs after the ALE, and the tobramycin concentration used to perform said ALE. The MICs for tobramycin and fosfomycin and the tobramycin concentrations used in the ALE were classified as "high" or "low" in order to be introduced in the program as binary code. The parental MIC for fosfomycin was considered "high" if the value was ≥ 64 μg/ml and "low" if the value was ≤ 48 μg/ml. The parental MIC for tobramycin was considered high if ≥ 2 μg/ml and low if ≤ 1.5 μg/ml. The tobramycin concentrations used in ALE assays were considered high if ≥ 2 μg/ml and low if ≤ 1 μg/ml. The MICs' fold change was calculated as the average of MICs of the strain evolved in presence of tobramycin, divided by the average of MICs of the same strain evolved without antibiotic. Resistome-related mutations in genes that were included: *oprD*, *pmrB*, *oprM*, *fusA*, *gyrB*, *mexA*, *mexX*, *mpl*, *gyrA*, *oprN*, *mexZ*, *ampC*, *mexS*, *mexY*, *armZ*, *mexB*, *ampR*, *parC* and *mexT*. TOB: tobramycin; FOF: fosfomycin.

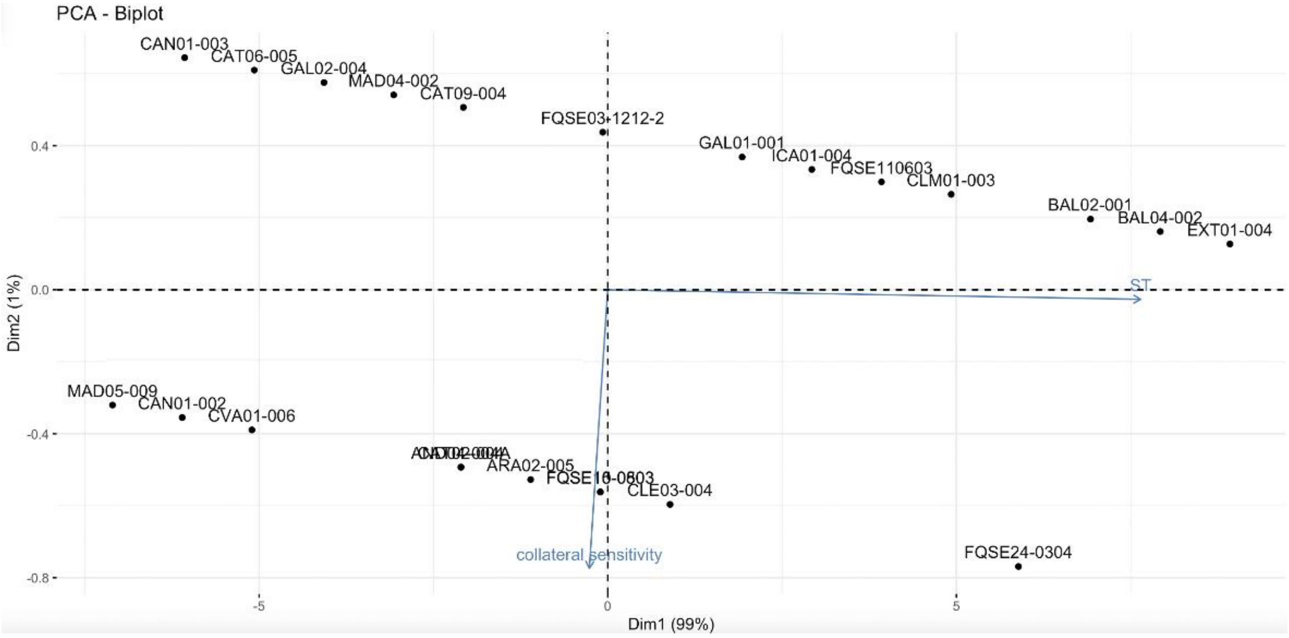

Figure S2. Principal component analysis of fosfomycin collateral sensitivity and sequence type (ST) as unique variables. The principal component analysis was done using as Dim1 the fosfomycin collateral sensitivity and the ST as Dim2. In the upper part of the biplot the strains that did not develop collateral sensitivity were plotted, whereas in the bottom there were the ones that developed said phenotype. The contribution to this division in two groups of the variable collateral sensitivity was 99%, while the one of the ST was 1%.
